# Serum galectin-3 and fibroblast growth factor-23 levels in relation with type 2 diabetes and cardiovascular risk

**DOI:** 10.5937/jomb0-50471

**Published:** 2025-01-24

**Authors:** Aleksandra Klisic, Sanja Gluscevic, Paschalis Karakasis, Jelena Kotur-Stevuljevic, Ana Ninic

**Affiliations:** 1 University of Montenegro-Faculty of Medicine, Podgorica, Montenegro; 2 Primary Health Care Center, Center for Laboratory Diagnostics, Podgorica, Montenegro; 3 Clinical Center of Montenegro, Department of Neurology, Podgorica, Montenegro; 4 Aristotle University of Thessaloniki, General Hospital "Hippokration", Second Department of Cardiology, Thessaloniki, Greece; 5 University of Belgrade, Faculty of Pharmacy, Department for Medical Biochemistry, Belgrade

**Keywords:** diabetes, inflammation, insulin resistance, biomarkers, dijabetes, inflamacija, insulinska rezistencija, biomarkeri

## Abstract

**Background:**

The clinical utility of galectin-3 and fibroblast growth factor 23 (FGF-23) needs to be further explored since previous studies show divergent results in relation to type 2 diabetes (T2D) and cardiovascular risk. Hence, the aim of this research was to explore galectin-3 and FGF-23 in relation to T2D, as well as to examine the potential association of these biomarkers with atherosclerotic cardiovascular disease (ASCVD) risk score in Montenegrin adults.

**Methods:**

A total of 35 T2D patients and 36 controls were consecutively enrolled. Serum galectin-3 and FGF-23 were determined by ELISA. The ASCVD risk score was calculated.

**Results:**

Higher serum galectin-3 levels were shown in T2D patients (p=0.016) in comparison with the control group. The increase in galectin-3 levels for 1 ng/mL showed an 8.5% higher probability of T2D occurrence (OR=1.085, p=0.015). FGF-23 levels did not differ between the control and the T2D group. Serum galectin-3 correlated with FGF23 (r=0.390, p=0.001). Both galectin-3 (r=0.306, p=0.010) and FGF-23 (r=0.332, p=0.005) correlated with ASCVD risk score in bivariate Spearman's correlation analysis, but these correlations were not retained in binary logistic regression analysis.

**Conclusions:**

Serum galectin-3 levels but not FGF-23 are higher in T2D patients. Serum galectin-3 correlated with FGF-23. Although both biomarkers were correlated with the ASCVD risk score, further statistical analysis did not confirm their independent associations with cardiovascular risk. Studies with a large sample size are needed to further explore this issue.

## Abstract (sr)

**Uvod:**

Klinička primena galektina-3 i fibroblastnog faktora rasta-23 (FGF-23) zahteva dodatna istraživanja s obzirom da su dosadašnje studije pokazale oprečne rezultate u odnosu na dijabetes tip 2 i kardiovaskularni rizik. S tim u vezi, cilj ovog istraživanja je bio da ispita galektin-3 i FGF-23 kod pacijenata sa dijabetesom tip 2, kao i da se ispita potencijalna povezanost ovih biomarkera sa skorom aterosklerotskog kardiovaskularnog rizika (ASCVD) u adultnoj crnogorskoj populaciji.

**Metode:**

Ukupno 35 pacijenata obolelih od dijabetesa i 36 ispitanika koji su činili kontrolnu grupu su konsekutivno uključeni u istraživanje. Serumske vrednosti galektina-3 and FGF-23 su merene ELISA metodom. ASCVD skor rizika je izračunat.

**Rezultati:**

Veče serumske vrednosti galektina-3 su zabeležene kod pacijenata obolelih od dijabetesa tip 2 (p=0,016) u poređenju sa kontrolnom grupom. Porast nivoa galektina-3 za 1 jedinicu pokazao je 8,5% veču vjerovatnoču za pojavu dijabetesa (OR=1,085, p=0,015). Vrednosti FGF-23 se nisu razlikovale među ispitivanim grupama. Serumske vrednosti galektina-3 su korelirale sa FGF-23 (p=0,390, p= 0,001 ). I galektin-3 ( p = 0,306, p= 0,01 0) i FGF-23 (p =0,332, p=0,005) su korelirali sa AscVd skorom rizika u Spearman-ovoj korelacionoj analizi, ali ove korelacije nisu zadržane u binarnoj logističkoj regresionoj analizi.

**Zaključak:**

Serumske vrednosti galektina-3, ali ne i FGF-23 su veče kod pacijenata obolelih od dijabetesa tip 2. Serumske vrednosti galektina-3 su korelirale sa FGF-23. Premda su oba biomarkera korelirala sa ASCVD skorom rizika, dublja statistička analiza nije potvrdila nezavisnu povezanost ovih biomarkera sa kardiovaskularnim rizikom. Potrebna su istraživanja na večem broju ispitanika da dodatno istraže ulogu ovih biomarkera u dijabetesu tip 2.

## Introduction

Diabetes mellitus is the most prevalent chronic disease worldwide. It is estimated that nearly 537 million adults have diabetes and this number is expected to increase up to 643 million by 2030 [1]. Nearly 90% of all diabetes cases are with type 2 diabetes (T2D) [2].

Cardiovascular disease (CVD) is the main cause of mortality and morbidity in individuals with diabetes [3].

Given the fact that T2D and CVD participate in common pathophysiological mechanisms, it is important to investigate novel inflammatory biomarkers in order to enable clearer insight into the multifactorial pathological processes of such chronic diseases [3] [4] [5]. These biomarkers could also provide a more reliable tool for the assessment of CVD risk in T2D patients [3] [4] [5].

In Montenegro, T2D is the third most frequent cause of years of life lived with disability, whereas CVD is the leading cause of death [6]. However, the lack of CVD risk assessment services are among the most prominent drawbacks in the primary care setting in Montenegro [6].

The estimation of the probability of an individual experiencing a major cardiovascular (CV) event in the next ten years, i.e. the calculation of CV risk score by validated algorithms, has shown to be one of the most valuable preventive strategies in adults, in order to make prompt therapeutic decisions [7]. Although there are several scores that have been validated in different ethnic groups, the ASCVD risk algorithm was shown to increase the accuracy of predicting CV events, and it makes the choice of strategies easier to adopt in primary prevention, according to the American College of Cardiology (ACC) and American Heart Association (AHA) guidelines [7]. In line with this, it was shown that subjects with ASCVD equal or higher than 7.5% had a 2-fold higher likelihood ofdying due to CVD in comparison with those at low risk after a follow-up of 17.7 years [8].

Galectin-3 and fibroblast growth factor 23 (FGF-23) are two biomarkers involved in processes of inflammation and fibrosis [9]. Galectin-3 is a 35 kDa protein and a part of the lectin family. It exhibits a wide diversity of properties, such as cellular adhesion, cellular growth, differentiation, proliferation, and apoptosis. It is also involved in atherosclerosis, by inducing proliferation and migration of endothelial cells and promoting chemoattraction of monocytes and macro phages, and clearance of neutrophils [10].

FGF-23 is a hormone secreted mainly by osteocytes and osteoblasts. It is a 32 kDa glycoprotein. Kidneys are the primary site of its action where it inhibits both, the reabsorption of phosphate and synthesis of 1,25-dihydroxyvitamin D (calcitriol). FGF-23 needs co-factor, klotho to exert its effects on the kidney and favors excessive discharge of phosphates in the urine. It exerts direct influence on the heart in a klotho-independent manner, thus contributing to the endothelial dysfunction and atherogenesis [11] [12].

However, the clinical utility of these biomarkers needs to be further explored, since previous studies show divergent results of galectin-3 [13] [14] [15] [16] [17] and FGF-23 [11] [18] [19] [20] in relation to parameters of glucose homeostasis (i.e., insulin resistance and glycated hemoglobin) and T2D. Moreover, although the majority of studies pointed out the significance of galectin-3 in CV setting [21], as for the FGF-23 the findings are rather controversial [12] [22] [23].

To the best of our knowledge, there are no studies that investigated galectin-3 and FGF-23 in relation to ACSVD risk in individuals with T2D. Hence, the aim of this research was to explore serum galectin-3 and FGF-23 in relation to T2D, as well as to examine the potential association of these biomarkers with ASCVD risk score in Montenegrin adults.

## Materials and methods

### Patients

A total of 35 T2D patients and 36 controls were consecutively enrolled in this cross-sectional study. The project was approved by the Institutional Ethics Committee and conducted according to the Helsinki Medical Declaration’s ethical principles, once the examinees signed an informed consent form.

The questionnaire (that contains questions related to demographic data, acute/chronic diseases, medication use, and lifestyle habits) was handed to each respondent. Anthropometric measurements and the venipuncture were performed the same morning.

Respondents were eligible to be included in the study if they voluntarily agreed to participate. The exclusion criteria for patients in the control group was a diagnosis of T2D or the use of antidiabetic medications. Patients with endocrine disorders other than T2D, and those with CVD, stroke, cancer, acute infection, autoimmune diseases, gout, pregnancy, as well as those who used anti-inflammatory drugs in the last month, were excluded from the research. Additionally, patients with estimated glomerular filtration rate <30 mL/min per 1.73 m^2^ and those with high-sensitivity C-reactive protein (hsCRP) 10.0 mg/L were also excluded.

The diagnosis of T2D was made according to the American Diabetes Association criteria [24].

### Methods

The blood sampling was provided in the morning after fasting of at least 8 hours. The samples were collected in serum separator/clot activator tubes for biochemistry analyses, except for HbA1c for which K2EDTA tubes were provided. All biochemical analyses were performed on Roche Cobas 6000 c501 chemistry analyzer (Roche Diagnostics GmbH, Mannheim, Germany) by standardized procedures. Serum galectin-3 and FGF-23 were determined by ELISA.

The ASCVD risk score was calculated [7]. The variables incorporated in the ASCVD calculation wereas follows: age, gender, race, smoking status, total cholesterol (TC), low density lipoprotein cholesterol (LDL-c), high density lipoprotein cholesterol (HDL-c), systolic blood pressure (SBP), diastolic blood pressure (DBP), hypertension treatment, use of statins, aspirin treatment, and history of diabetes.

The blood pressure was measured for each participant. Body weight, body height, waist (WC) and hip circumference (HC) were obtained in the morning hours, whereas body mass index (BMI) was calculated.

### Statistical analysis

A data distribution was tested using the Shapiro Wilk test in SPP Statistics ver. 21 (IBM, New York,USA), as well as all statistical analysis. Gaussian distributed variables were presented as mean ± standard deviation (SD) and tested using Student’s t-test. Comparisons between non-Gaussian distributed data were performed using the Mann-Whitney test. Those data were given as the median and interquartile range. Categorical data, presented as absolute and relative frequencies, were compared using the Chi-square test for contingency tables. Spearman’s correlation analysis was performed between HbA1c, galectin-3, and FGF-23 levels and other clinical characteristics of all subjects. Correlation coefficient (ρ) was used to present correlations between tested markers. Associations between T2D, galectin-3, and FGF-23 were further tested using binary regression analysis. Potential independent associations between them were tested by multivariate binary regression analysis where covariates were those which had significant r with clinical and laboratory data. Data were given as odds ratio (OR) and 95% Confidence interval (CI). The explained variation in T2D was given with Nagelkerke R^2^. P levels less than 0.05 were considered statistically significant in all tests.

## Results

Clinical characteristics of the examined population are presented in Table 1. Gender was not evenly distributed in the examined groups. More women were in the control group, but more men were in the T2D group. T2D patients had higher BMI and WC than controls. Patients with T2D used antidiabetic therapy. However, as it would be expected by inclusion criteria, control group did not use antidiabetic medications.

**Table 1:** Basic demographic characteristics of examined population. Data are presented as mean±SD and compared using Student’s t-test for two independent samples.<br>*Skewed distributed data are presented as median (interquartile range) and compared using Mann-Whitney U test.<br>Categorical variables are presented as absolute and relative frequencies and compared using Chi-square test.

	Control group	Type 2 diabetes	*p*
**N (male/female)**	36 (12/24)	35 (22/13)	**0.013**
**Age, years**	61±10	65±11	0.166
BMI, kg/m^2*^	28 (25–30)	31 (28–31)	**0.006**
**WC, cm**	95±9	104±11	**<0.001**
HC, cm*	107 (103–109)	107 (105–114)	0.287
SBP, mmHg*	138 (127–173)	140 (129–159)	0.546
DBP, mmHg*	86 (77–92)	81 (77–88)	0.254
**Insulin, N (%)**	0 (0)	8 (23)	**0.002**
**Antihyperglycemics, N (%)**	0 (0)	29 (83)	**<0.001**
Diabetes duration, years*	–	3 (1–10)	–
**Smoking habits, N (%)**	14 (39)	10 (29)	0.358
**Antihypertensives, N (%)**	26 (72)	30 (86)	0.164

Parameters of glucose homeostasis, including glucose and HbA1c, were higher in T2D patients than controls. Total cholesterol, LDL-c, and TG were, also, higher in T2D patients than in controls. However, HDL-c was lower in T2D patients. We observed significantly higher creatinine and uric acid levels, as well as galectin-3 levels in T2D patients. Higher ASCVD risk was also evident in patients with T2D (Table 2) as compared with controls. Serum FGF-23 levels did not differ between the tested groups.

**Table 2:** Laboratory and clinical parameters of examined population. Data are presented as mean±SD and compared using Student’s t-test.<br>*Skewed distributed data are presented as median (interquartile range) and compared using Mann-Whitney *U* test.

	Control group	Type 2 diabetes (T2D)	*p*
Glucose, mmol/L	5.9±0.6	8.6±3.3	**<0.001**
HbA1c, %	5.7±0.4	7.7±1.9	**<0.001**
Total cholesterol, mmol/L*	6.1 (5.3–6.6)	5.3 (4.9–6.0)	**0.032**
HDL-c, mmol/L*	1.4 (1.2–1.7)	1.2 (1.0–1.4)	**0.006**
LDL-c, mmol/L*	3.8 (3.0–4.2)	3.1 (2.7–3.1)	**0.024**
TG, mmol/L*	1.5 (1.2–2.2)	1.9 (1.6–2.8)	**0.034**
Total bilirubin, μmol/L*	8.8 (6.5–11.1)	8.6 (6.0–10.8)	0.931
Creatinine, μmol/L*	69 (59–81)	80 (67–92)	**0.010**
Uric acid, μmol/L	273±60	337±104	**0.002**
HsCRP, mg/L*	1.3 (0.7–2.8)	1.7 (0.8–3.2)	0.451
Galectin-3, ng/mL*	17.79 (12.44–24.18)	21.73 (15.77–36.11)	**0.016**
FGF-23, ng/mL*	0.92 (0.87–1.10)	0.95 (0.85–1.26)	0.995
ASCVD, %	12 (4–28)	35 (16–54)	**<0.001**

Further, we performed a correlation analysis between the glucose homeostasis marker HbA1c, galectin-3 and FGF-23 and other examined markers. HbA1c displayed positive correlations with WC, glucose, TG, and ASCVD risk. However, negative correlations were evident between HbA1c and total cholesterol, HDL-c and LDL-c (Table 3). Galectin-3 showed positive correlations with SBP, FGF-23, and ASCVD. Also, positive correlations were evident between FGF-23 and SBP and ASCVD risk score (Table 3).

**Table 3:** Bivariate Spearman’s correlation analysis of HbA1c, galectin-3, FGF-23 and other clinical markers.

	HbA1c, %		Galectin-3		FGF-23	
	ρ	p	ρ	p	ρ	p
Age, years	0.120	0.320	0.076	0.528	0.093	0.442
BMI, kg/m	0.222	0.067	-0.033	0.786	-0.075	0.539
WC, cm	**0.362**	**0.002**	0.113	0.357	0.039	0.752
HC, cm	0.109	0.373	-0.070	0.565	-0.068	0.580
SBP, mmHg	0.114	0.344	**0.293**	**0.013**	**0.621**	**<0.001**
DBP, mmHg	0	0.998	0.031	0.795	0.072	0.553
Glucose, mmol/L	**0.756**	**<0.001**	-0.026	0.827	0.016	0.896
HbA1c, %	-	-	0.172	0.151	0.082	0.496
TC, mmol/L	**-0.284**	**0.017**	0.017	0.888	0.064	0.594
HDL-c, mmol/L	**-0.341**	**0.004**	0.033	0.785	0.018	0.883
LDL-c, mmol/L	**-0.269**	**0.023**	0.014	0.906	0.116	0.336
TG, mmol/L	**0.273**	**0.021**	-0.012	0.919	-0.011	0.927
Total bilirubin, μmol/L	-0.007	0.954	0.098	0.414	0.197	0.100
Creatinine, μmol/L	0.214	0.074	0.126	0.296	0.148	0.219
Uric acid, μmol/L	0.121	0.314	0.015	0.901	-0.038	0.752
HsCRP, mg/L	0.127	0.293	0.086	0.476	0.017	0.888
Galectin-3, ng/mL	0.082	0.497	-	-	**0.390**	**0.001**
FGF-23, ng/mL	0.082	0.377	**0.390**	**0.001**	**-**	**-**
ASCVD, %	**0.391**	**0.001**	**0.306**	**0.010**	**0.332**	**0.005**

Regression analysis determined a positive association between galectin-3 and T2D (Table 4). As galectin-3 concentration rose for 1 ng/mL the probability of T2D increased by 7.3% (OR=1.073, p=0.008). Galectin-3 levels were able to explain 19.9% variation in T2D presence. On the contrary, no significant association between FGF-23 and T2D was evident.

**Table 4:** Odds ratios (OR) after univariate and multivariate binary logistic regression analysis for galectin-3 and FGF-23 predicting T2D. Model: continuous variables: WC, HDL-C, TG and categorical variable: sex.

Predictors	Unadjusted OR (95%CI)	*p*	Nagelkerke *R^2^*
**Galectin-3**	1.073 (1.019–1.130)	**0.008**	0.199
**FGF-23**	0.935 (0.700–1.248)	0.647	0.004
Model	Adjusted OR (95%CI)	*p*	Nagelkerke *R^2^*
**Galectin-3**	1.085 (1.016–1.159)	**0.015**	0.458

To adjust for covariates that were correlated with galectin-3 in Spearman’s correlation analysis, multivariate binary regression analysis was performed. Interestingly, participants with galectin-3 levels that increased for 1 ng/mL have an 8.5% higher probability of T2D occurrence (OR=1.085, p=0.015). The explained variation in T2D by this model was 45.8% (Table 4).

Neither galectin-3, nor FGF-23 were associated with higher ASCVD risk when performed univariate binary logistic regression analysis (Table 5).

**Table 5:** Odds ratios (OR) after binary logistic regression analysis for galectin-3 and FGF-23 predicting ASCVD risk.

Predictors	Unadjusted<br>OR (95%CI)	p	Nagelkerke<br>R^2^
Galectin-3	1.035<br>(0.982–1.090)	0.202	0.046
FGF-23	66.977<br>(0.820–5471.026)	0.061	0.159

## Discussion

The main finding of this study shows higher serum galectin-3 levels in T2D patients. An increase in galectin-3 levels for 1 ng/mL showed 8.5% higher probability for T2D occurrence (OR=1.085, p=0.015). However, serum FGF-23 did not differ between control and T2D group. We have also shown for the first time the correlation between serum galectin-3 and FGF-23 levels. Moreover, both galectin-3 and FGF-23 correlated with ASCVD score in bivariate Spearman’s correlation analysis, but these correlations were not retained in binary logistic regression analysis.

Previous studies show divergent results regarding serum galectin-3 levels in relation to parameters of glucose homeostasis. An inverse correlation between serum galectin-3 levels and insulin resistance [14] and HbA1c in T2D subjects [15] was shown. On the contrary, a positive correlation between serum galectin-3 and HbA1c was demonstrated by other authors [17]. Our results are in line with a recent study by Echouffo-Tcheugui et al. [16] that showed higher serum galectin-3 levels in patients with T2D. Also, Vora et al. [13] showed a positive association between serum galectin-3 levels and incident T2D. The authors of this large sample size longitudinal study demonstrated that serum galectin-3 levels were associated with several pro-inflammatory cytokines and parameters of insulin secretion [i.e., Cpeptide/homeostasis model assessment of insulin resistance (HOMA-IR) ratio and C-peptide] [13]. Since the authors of the latter study did not confirm the association between galectin-3 and HOMA-IR, they presumed that the role of galectin-3 in T2D could not be attributed to its involvement in signaling pathways related to insulin resistance, but rather to the pathways included in insulin secretion. In line with this, Petrovic et al. [25] showed that galectin 3-overexpression enhances oxidative stress and facilitates the damage and apoptosis of pancreatic β-cells, thus affecting glucose metabolism in high-fat diet mice.

Concerning FGF-23, we did not observe any difference between subjects with T2D and the controlgroup. Lower serum FGF-23 levels were found in women with gestational diabetes mellitus [18] and an insulin-resistant obese adolescent population [19], whereas some other studies demonstrated higher FGF-23 in T2D [26]. Hanks et al. [20] suggested that renal function might mediate the relationship between FGF-23, inflammation, and insulin resistance since they recorded positive associations between FGF-23, HOMA-IR and several obesity-related pro-inflammatory cytokines only in subjects without chronic kidney disease (CKD), but not among those with CKD.

Insulin was shown to suppress FGF-23 by stimulating the phosphoinositide 3-kinase/protein kinase B/Akt signaling transcription factor forkhead box protein O1 [27]. The treatment with insulin in mice and cell culture diminished FGF-23 gene expression [27]. Also, higher FGF-23 levels were recorded in insulin-deficient mice, whereas the insulin treatment normalized FGF-23 levels [27]. Such findings support the notion that insulin could lower FGF-23, independent of changes in kidney function or inflammation.

To the best of our knowledge, we are the first to show the correlation between galectin-3 and FGF-23. These two pro-inflammatory biomarkers are indicators of fibrosis and CV remodeling [9]. Previous studies were not convincing regarding the role of FGF-23 in CVD, thus suggesting only a minor contribution of FGD-23 as predictors of CVD outcomes [12] [28]. The CARDIA study that included 3151 participants showed no relationship with CVD after adjustment for confounding factors, such as diabetes, BMI, SBP, smoking status, and medication use [28].

Kurpas et al. [22] pointed out that the importance of FGF-23 as a risk factor for CVD should not be overstated in T2D patients, showing no correlation between FGF-23 and fasting glucose, postprandial glucose, and HbA1c values. On the other hand, some other authors demonstrated that plasma FGF-23 levels predict adverse CV outcomes in patients with coronary artery disease (CAD) and T2D, but not in those without T2D [23].

In a recent meta-analysis, galectin-3 was shown to be one of the risk factors for CVD [21]. Galectin-3 was also shown to be a reliable biomarker in atrial fibrillation [29] and obstructive sleep apnea [30]. However, a recent study that examined subjects with hepatic steatosis showed an inverse relationship between severity the of CAD and galectin-3 [31]. In our study, we did not confirm the independent relationship between serum galectin-3 levels and the ASCVD risk score. The possible explanation for such finding may be in part due to the small sample size of our study. Apart from the small sample size, this study has several other limitations, such as its cross-sectional nature, which does not enable us to evaluate the causality between investigated biomarkers and T2D. We were also limited to measure serum klotho levels, vitamin D levels, as well as to assess dietary phosphate intake since these variables could significantly affect serum FGF-23 levels [26]. Therefore, longitudinal studies with a large sample size that will include mentioned confounding factors are needed to elucidate the role of galectin-3 and FGF-23 in T2D and increased CV risk.

## Conclusion

Serum galectin-3 levels, but not FGF-23, are higher in T2D patients. Serum galectin-3 is correlated with FGF-23. Although both biomarkers were correlated with the ASCVD risk score, a further statistical analysis did not confirm their independent associations with CV risk. Studies with a large sample size are needed to further explore this issue.

## Dodatak

### Acknowledgments

This work was financially supported in part by a grant from the Ministry of Education, Science and Innovation, Montenegro and the Ministry of Education, Science and Technological Development, Republic of Serbia (project number 451-03-65/2024-03/200161).

### Author Contributions

All authors contributed to the study conception and design. Material preparation, data collection and laboratory analyses were performed by AK and JKS. Statistical analysis was performed by AN. The first draft of the manuscript was written by AK and all authors commented on previous versions of the manuscript. All authors read and approved the final version of the manuscript.

### Conflict of interest statement

All the authors declare that they have no conflict of interest in this work.
